# Long-Term Prognosis of Unstable Angina: A Ten-Year Follow-Up Study Comparing Clinical Outcomes with Myocardial Infarction

**DOI:** 10.3390/medicina62101857

**Published:** 2026-09-25

**Authors:** Raquel Romojaro-López, Juan Gordo-Claros, Alfredo Bardají, Mercedes Vicente-de-Vera-Bueno, Itsaso Agirre-Bartolomé, José Luis Ferreiro

**Affiliations:** 1 Departament of Cardiology, Joan XXIII University Hospital, 43005 Tarragona, Spain; raquel.romojaro@estudiants.urv.cat (R.R.-L.); juan.gordo@estudiants.urv.cat (J.G.-C.); mvicentedevera.hj23.ics@gencat.cat (M.V.-d.-V.-B.); iagirre.hj23.ics@gencat.cat (I.A.-B.); jferreiro.hj23.ics@gencat.cat (J.L.F.); 2 Department of Medicine and Surgery, Universitat Rovira i Virgili, 43005 Tarragona, Spain; 3 Institut de Recerca Biomèdica Catalunya Sud (IRB CatSud), 43005 Tarragona, Spain; 4 Cardiology Service, Tarragona Joan XXIII University Hospital, Rovira Virgili University, IISPV, Spain, Calle Dr Mallafré Guasch 4, 43005 Tarragona, Spain

**Keywords:** unstable angina, acute coronary syndrome, prognosis

## Abstract

*Background and Objectives*: Unstable angina has traditionally been considered the least severe acute coronary syndrome presentation, but ten-year prognostic data remain limited. We compared the clinical characteristics and ten-year prognosis of unstable angina versus non-ST-segment elevation myocardial infarction and ST-segment elevation myocardial infarction. *Materials and Methods*: This retrospective study included consecutive patients older than eighteen years admitted with suspected acute coronary syndrome to a Spanish tertiary hospital between January 2009 and December 2015. Ten-year survival and clinical outcomes were evaluated retrospectively through long-term tracking of electronic medical records and official regional death registries. *Results*: Of 3029 patients (10.6% with unstable angina, 45.0% with non-ST-segment elevation myocardial infarction, and 44.4% with ST-segment elevation myocardial infarction), those with unstable angina had a median age of 66.0 years (vs. 67.0 and 62.0 years in the other cohorts, respectively; *p* < 0.001) and a significantly higher prevalence of prior myocardial infarction (31.5% vs. 21.9% and 11.4%, respectively; *p* < 0.001). However, unstable angina presented lower-risk acute profiles (Killip class >I: 2.5% versus 17.5% and 20.7%; *p* < 0.001; median GRACE score: 89.9 versus 107.6 and 113.3; *p* < 0.001) and 0% in-hospital mortality (versus 3.3% and 5.4%; *p* < 0.001). At ten years, cumulative mortality reached 23.4% in unstable angina, converging with ST-segment elevation myocardial infarction (29.1%; *p* > 0.05) but remaining lower than non-ST-segment elevation myocardial infarction (35.3%; *p* < 0.05). In multivariable Cox analysis, unstable angina was not independently associated with ten-year mortality (overall *p* = 0.066; vs. STEMI *p* = 0.743; vs. NSTEMI *p* = 0.133). *Conclusions*: Despite favorable acute stabilization and the absence of in-hospital mortality, the survival advantage of unstable angina over ST-segment elevation myocardial infarction appears to attenuate over a ten-year period. This trend toward mortality convergence is likely associated with the baseline comorbidity burden, highlighting the clinical need for intensive secondary prevention.

## 1. Introduction

Acute coronary syndrome (ACS) remains the leading cause of cardiovascular morbidity and mortality worldwide [1]. It encompasses a broad clinical spectrum comprising ST-segment elevation myocardial infarction (STEMI), non-ST-segment elevation myocardial infarction (NSTEMI), and unstable angina (UA), with the latter two collectively referred to as non-ST-segment elevation acute coronary syndromes (NSTE-ACS) [2]. Across the ACS spectrum, STEMI involves transmural necrosis with ST elevation, NSTEMI subendocardial necrosis with positive biomarkers, and unstable angina troponin-negative ischemia [3].

Numerous studies evaluating short- and intermediate-term outcomes (i.e., in-hospital, 30-day, and 1-year follow-up) have consistently demonstrated lower mortality rates among patients with UA compared to those with STEMI and NSTEMI [4,5]. Most notably, the widespread clinical integration of high-sensitivity cardiac troponin assays has driven a diagnostic shift from UA to NSTEMI by identifying cases of minor myocardial injury previously classified as UA [6,7]. This epidemiological shift has resulted in a contemporary UA population that is better defined but still not well characterized.

Furthermore, evidence regarding outcomes beyond the first-year post-index event remains sparse [8,9]. The few studies reporting long-term prognostic data are hindered by heterogeneous patient cohorts, varying study designs, and inconsistent data collection methods, thereby limiting both the comparability of their findings and their generalizability to contemporary clinical practice [10,11].

We hypothesized that the early prognostic advantage observed in patients with UA during hospitalization and the initial year post-event may attenuate over extended follow-up. Therefore, the primary objective of this study was to compare the long-term prognosis of patients presenting with UA against those with NSTEMI and STEMI, determining whether the favorable short- and intermediate-term outcomes historically associated with UA are sustained longitudinally.

## 2. Materials and Methods

### 2.1. Study Design and Patient Population

This is an observational, longitudinal, and retrospective study including patients older than 18 years admitted consecutively with suspected acute coronary syndrome between January 2009 and December 2015 at a tertiary university hospital in Spain. The study was conducted and is reported in accordance with the Strengthening the Reporting of Observational Studies in Epidemiology (STROBE) guidelines for cohort studies. The systematic review of medical records and all available information allowed the classification of patients into the following categories: definitive acute coronary syndrome, chest pain, and other diagnoses. Acute coronary syndrome was defined as STEMI, NSTEMI, and UA, in accordance with the European Society of Cardiology guidelines applicable at the time of diagnosis [12,13]. All diagnoses of UA were reviewed by a senior investigator (AB). UA was defined as the presentation of typical coronary chest pain without evidence of myocardial necrosis. Cardiac troponin I was determined using the TnI-Ultra assay on the Advia Centaur platform (Siemens Healthineers, Erlangen, Germany). In accordance with clinical guidelines, cardiac troponin I status was evaluated as a categorical diagnostic criterion (>39 ng/L for myocardial infarction vs. ≤39 ng/L for unstable angina) rather than registered as a continuous quantitative variable for all patients in the registry database. The ischemic origin of the symptoms was supported by a documented history of ischemic heart disease, a non-invasive imaging test demonstrating myocardial ischemia, or a cardiac catheterization revealing significant coronary artery lesions. Beyond considering only the index admission in cases of repeated hospitalizations during the study period, exclusion criteria comprised cases of chest pain where an acute coronary syndrome was not confirmed, as well as alternative conditions, including Takotsubo cardiomyopathy, acute aortic syndrome, and myocarditis, among other diagnoses.

### 2.2. Clinical Variables Studied

Demographic variables, cardiovascular risk factors, and relevant medical history were collected through a systematic review of medical records. Regarding clinical presentation, the chief complaint, electrocardiographic findings, physical examination parameters, and Killip-Kimball class at admission were recorded. Baseline laboratory data were extracted, including the elevation of biomarkers of myocardial injury, and the estimated glomerular filtration rate (eGFR) was calculated using the Modification of Diet in Renal Disease-4 (MDRD-4) equation. Additionally, the performance of complementary cardiac investigations (echocardiography, ischemia testing, and coronary angiography) was documented, detailing the anatomical extent of the disease (number of diseased vessels) and the need for percutaneous coronary intervention. During 2009–2015, coronary computed tomography angiography was not routinely performed; instead, stabilized low-to-intermediate risk troponin-negative unstable angina patients underwent pre-discharge exercise testing per contemporary guidelines. Finally, incident in-hospital complications and the clinical and vital status at discharge were recorded.

### 2.3. Primary and Secondary Endpoints

The primary endpoint of the study was all-cause mortality at 10 years of follow-up in patients with UA, compared with those with STEMI and NSTEMI. Secondary endpoints included readmission for heart failure, readmission for myocardial infarction, and the composite endpoint of death, readmission for myocardial infarction, or heart failure readmission (major adverse cardiovascular events [MACE]) during the same period. Analyses were also reported for the index admission, and at 1 and 5 years of follow-up. Follow-up events were extracted from the patients’ electronic medical records and death registries. Patients lost to follow-up immediately following hospital discharge due to returning to their countries of origin were treated as censored observations at time zero in the survival analysis.

### 2.4. Statistical Analysis

Quantitative variables are expressed as medians and interquartile ranges (25th–75th percentiles). Categorical variables are expressed as frequencies and percentages. The comparison of quantitative variables among the three groups was analyzed using the Kruskal–Wallis test. The association between categorical variables and the groups was assessed using contingency table analysis and the chi-square test. In both instances, when statistically significant differences were observed, post hoc pairwise comparisons with Bonferroni correction were performed to identify which groups differed regarding their medians or proportions. Survival analysis across the three diagnostic groups was conducted using the Kaplan–Meier method, and survival curves were compared using the log-rank test. Cox regression was used for mortality models, with proportional hazards verified via log-log plots and Schoenfeld residuals. A Fine-Gray model calculated subdistribution hazard ratios (sHR) for readmissions, treating death as a competing risk. Multivariable Cox models included variables with univariate *p* < 0.10, forcing age and sex. The model adjusted for age, sex, diabetes, Killip > I, eGFR, GRACE score, depressed ejection fraction, angioplasty, and ACS subtype, using backward elimination (*p*-out > 0.10). Complete-case analysis was used for missing data (<5%), and patients lost post-discharge were censored at time zero. Given the 10-year follow-up, age and eGFR were included alongside the GRACE score to capture long-term risk; however, to address potential multicollinearity, a prespecified sensitivity analysis excluded GRACE components (age, Killip, and eGFR). Results are reported as HR and sHR with 95% CIs, with significance set at *p* < 0.05. All statistical analyses were performed using IBM SPSS Statistics, version 32.0 (IBM Corp., Armonk, NY, USA). Advanced data visualization was conducted within the R statistical environment, utilizing the ‘survival’ and ‘survminer’ packages to generate the high-resolution Kaplan–Meier estimates and the adjusted hazard ratio forest plots.

### 2.5. Ethics Approval and Consent to Participate

The study was approved by the Clinical Research Ethics Committee (Comitè Ètic d’Investigació Clínica) of the Hospital Universitari de Tarragona Joan XXIII. Written informed consent was waived due to the retrospective nature of the analysis and the lack of any clinical intervention on the patients.

## 3. Results

Between January 2009 and December 2015, 3769 patients were admitted with suspected ACS. After exclusions, the final cohort comprised 3029 episodes: 1345 (44.4%) with STEMI, 1363 (45.0%) with NSTEMI, and 321 (10.6%) with UA (Figure 1).

### 3.1. Baseline Characteristics and In-Hospital Data

The median age of the cohort was 65 years, with a predominance of male patients (Table 1). Patients with UA had a median age of 66.0 years, which was numerically higher than those with STEMI (62.0 years) and closer to those with NSTEMI (67.0 years), although these pairwise differences did not reach statistical significance after Bonferroni correction. Patients with UA exhibited the highest burden of prior cardiovascular disease, including previous myocardial infarction, prior revascularization, and a greater prevalence of traditional cardiovascular risk factors compared to the STEMI and NSTEMI groups. Consequently, baseline secondary prevention pharmacotherapy (aspirin, P2Y12 inhibitors, beta-blockers, and statins) was significantly more prevalent in the UA cohort prior to admission (Table 1).

Chest pain was the predominant presentation across all groups, particularly in UA (Table 2). Despite their heavy comorbidity, patients with UA presented with a significantly lower risk profile during the acute phase, evidenced by lower median GRACE scores, fewer instances of Killip class > I, and a preserved left ventricular ejection fraction in the vast majority of cases (Table 2).

Regarding invasive management, patients with UA underwent coronary angiography less frequently than those with STEMI or NSTEMI; however, among those evaluated, the prevalence of multivessel coronary disease was comparable between UA and NSTEMI (Table 2).

In-hospital mortality was 0% in the UA group, contrasting sharply with both myocardial infarction cohorts (Table 3). At discharge, prescription rates for guideline-directed medical therapy in surviving UA patients were high and comparable to those with NSTEMI (Table 3).

### 3.2. Long-Term Survival and All-Cause Mortality

Absolute event rates increased progressively over time across all cohorts (Table 4). At 1 year, all-cause mortality and MACE rates were significantly lower in the UA group compared to STEMI and NSTEMI (Figure 2, Table 4). While this survival advantage persisted at 5 years, extended follow-up to 10 years revealed an attenuation of this gap: cumulative 10-year mortality in the UA cohort reached 23.4%, showing no statistically significant difference compared with the STEMI group (29.1%), though remaining lower than NSTEMI (35.3%) (Table 4).

### 3.3. Competing Risks Analysis for Long-Term Recurrences

Because death from any cause acts as a competing event for non-fatal clinical endpoints, a multivariate Fine-Gray competing risks regression model was employed to evaluate 10-year readmissions. Using UA as the reference group, the subdistribution hazard ratio (sHR) for heart failure readmission in patients with STEMI was 0.78 (95% CI 0.54–1.13; *p* = 0.190). In the same model, NSTEMI was not significantly associated with a higher risk of heart failure readmission (sHR 1.12, 95% CI 0.79–1.59; *p* = 0.520). Regarding recurrent myocardial infarction, the sHR for STEMI compared to UA was 0.90 (95% CI 0.63–1.28; *p* = 0.570). Conversely, patients with NSTEMI exhibited a trend toward an increased risk for recurrent ischemic events, although it did not reach statistical significance (sHR 1.36, 95% CI 0.97–1.92; *p* = 0.075).

### 3.4. Predictors of 10-Year Mortality

In the unadjusted univariate Cox regression analysis, all evaluated baseline characteristics and clinical variables demonstrated a statistically significant association with 10-year mortality (all *p* < 0.05).

It is important to note that Killip class > I at admission lost its independent prognostic value when adjusted for the comprehensive GRACE score and other comorbidities. Regarding the type of acute coronary syndrome, although it was significantly associated with mortality in the unadjusted analysis, it lost its independent predictive value after multivariate adjustment (overall *p* = 0.066). Using unstable angina (UA) as the reference group, neither STEMI (adjusted HR 1.048, 95% CI 0.791–1.389; *p* = 0.743) nor NSTEMI (adjusted HR 1.216, 95% CI 0.942–1.570; *p* = 0.133) were independently associated with an increased long-term mortality risk (Figure 3). To address the potential multicollinearity of including the GRACE score alongside its constituent variables, a sensitivity analysis was performed excluding age, Killip class, and eGFR. This model yielded fully consistent results regarding the primary endpoint: using unstable angina as the reference, neither STEMI (adjusted HR 0.765; *p* = 0.055) nor NSTEMI (adjusted HR 1.000; *p* = 0.998) were independently associated with increased 10-year mortality, confirming the robustness of the primary model.

## 4. Discussion

The present study evaluates the 10-year prognostic trajectory of patients with UA compared to those with STEMI and NSTEMI, revealing a significant clinical paradox. Our principal finding is that the profound survival advantage observed in UA patients during the acute phase and early follow-up is not sustained over the long term. Although UA patients present with the lowest acute clinical severity—evidenced by having the lowest risk scores on the GRACE scale—they paradoxically exhibit the highest baseline burden of chronic cardiovascular comorbidities, prior ischemic events, and multivessel disease.

The GRACE score is a well-established tool for predicting acute and post-discharge outcomes in acute coronary syndromes. In our cohort, the lower median GRACE score (89.9) of UA patients successfully reflected their low acute-phase risk and zero in-hospital mortality [14]. This residual risk is heavily driven by a high baseline comorbidity burden, a clinical profile that closely mirrors other major registries. In the APACE and High-STEACS cohorts, UA patients had a very high prevalence of pre-existing coronary artery disease (73% and 76%, respectively) [4], while in the Spanish ACHILLES registry, they presented with a heavier baseline risk factor burden than NSTEMI patients [15]. This pattern, where cumulative chronic comorbidity outweighs the acute presentation phenotype, is also observed in contemporary cohorts of critically ill cardiac patients [8,9,16], where long-term survival was determined predominantly by age and comorbidity burden (e.g., Charlson Comorbidity Index, atrial fibrillation, and prior stroke) rather than the index ACS subtype itself. Crucially, once adjusted for baseline disparities in acute presentation, the 10-year mortality risk of UA does not differ significantly from that of STEMI [17].

### 4.1. Diagnostic Accuracy and the Impact of Advanced Troponin Assays

The rigorous prognostic evaluation of UA fundamentally relies on an accurate initial diagnosis to distinguish true ischemia from non-specific chest pain, which carries a benign prognosis [11]. In our study, UA was strictly defined using established clinical criteria requiring typical symptoms and objective evidence of underlying coronary artery disease (such as positive imaging or angiographic lesions) to ensure the cohort represents true myocardial ischemia. Over recent decades, the adoption of fourth-generation and high-sensitivity cardiac troponin assays has driven an epidemiological shift [6,18,19]. In our cohort, biomarker assessment was consistently performed using a fourth-generation assay (TnI-Ultra from Siemens, Advia Centaur), which exhibits an analytical performance and sensitivity profile closely mirroring that of contemporary high-sensitivity troponin assays [20]. This definition of a high-risk biomarker-negative cohort is strongly supported by SWEDEHEART registry data, where patients with strictly defined UA exhibited a nearly 3-fold higher adjusted risk of major adverse cardiovascular events at 1 year (HR 2.97, 95% CI 1.30–6.78) compared to healthy controls [19]. The fact that this biomarker-negative cohort demonstrates a 10-year risk of non-fatal ischemic events comparable to STEMI underscores that the absence of detectable myocardial necrosis at presentation does not equate to a benign long-term coronary pathophysiology [21,22].

### 4.2. Loss of Independent Prognostic Value and Competing Risks

In the univariate Cox analysis, UA showed a protective trend compared to STEMI. However, after multivariate adjustment for age, diabetes, renal function, GRACE score, and left ventricular ejection fraction, this association lost significance and its direction was reversed. This shift indicates that the apparent unadjusted prognostic advantage of UA is entirely explained by its more favorable clinical profile during the acute phase (lower Killip class, better ejection fraction, lower GRACE score) rather than an inherently more benign course of the underlying coronary disease. Once these acute severity differences are statistically adjusted, UA ceases to behave as an entity with a better prognosis compared to STEMI.

Furthermore, competing risk analyses using the Fine-Gray model reveal that UA’s 10-year risk of non-fatal recurrences, including heart failure and recurrent myocardial infarction, is statistically comparable to STEMI survivors. Although this trajectory aligns UA with a high-risk chronic coronary syndrome profile, this apparent comparability should be interpreted with caution. Rather than reflecting an equivalent acute pathophysiological severity, this long-term risk is heavily driven by the higher baseline comorbidity and the recurrent (non-incident) nature of the coronary disease in the UA cohort. This high burden of recurrence is supported by prospective clinical data showing that even strictly adjudicated, troponin-negative UA carries a 1-year recurrent MI rate of 1.9%, which is statistically comparable to the 2.4% observed in NSTEMI [5]. Nevertheless, these comparable recurrence rates, both in our model and in external series, predominantly reflect the chronic, cumulative secondary vulnerability of a pre-selected, multi-recurrent patient population rather than an inherently aggressive acute phenotype.

### 4.3. Invasive Management and the Treatment-Risk Paradox

Despite having a significant coronary artery disease burden comparable to NSTEMI [23], patients with UA systematically received a less invasive revascularization approach in several clinical series, as reflected in lower rates of angioplasty [5]. In our study, 69.5% of patients with unstable angina underwent invasive coronary angiography during the index admission, leaving 30.5% managed conservatively without catheterization. This lower invasive rate reflects real-world clinical practice during the study period, where patients presenting with non-elevated biomarkers, complete symptom resolution, advanced age, severe non-cardiac comorbidities, or reassuring non-invasive stress testing were frequently managed with conservative medical therapy. Insofar as revascularization provides a prognostic benefit, the lower rate of angioplasty observed in UA could be limiting their potential long-term benefit [24]. This therapeutic mismatch represents a classic manifestation of the ‘treatment-risk paradox’ documented in major clinical registries, where high-risk patients are paradoxically less likely to undergo routine invasive revascularization strategies [24]. In our multivariable model, percutaneous coronary intervention was independently associated with lower 10-year mortality. However, given the observational design of our registry, this finding reflects a prognostic association rather than a confirmed treatment effect, as residual confounding by indication cannot be excluded. Breaking this clinical bias by implementing aggressive secondary prevention [2,25] and revascularization could significantly improve long-term outcomes in UA.

### 4.4. Database Design, Strengths, and Limitations

Our database was modeled on established registries such as SWEDEHEART [26]. Unlike the MINAP database, which prioritizes acute care quality and lacks long-term follow-up data [27], our model provides comprehensive insight into each patient’s status at admission alongside personal identification numbers, enabling long-term follow-up of both mortality and morbidity. Several limitations of our study must be acknowledged. First, the retrospective, single-center design of our registry may limit the generalizability of the findings to other healthcare settings with different interventional resources. Despite extensive adjustments, the observational nature of our data means that residual confounding from unmeasured clinical variables cannot be entirely excluded. Second, baseline characteristics among the three ACS cohorts were markedly unbalanced; patients with unstable angina presented with a significantly higher prevalence of prior cardiovascular disease and chronic risk factors but lower acute hemodynamic severity compared to infarction cohorts. Consequently, crude unadjusted outcome comparisons between groups must be interpreted with extreme caution. To mitigate this baseline imbalance, we performed multivariable Cox proportional hazards modeling and Fine-Gray competing risk regression to control for independent clinical confounders. Third, the sample size of our unstable angina cohort was considerably smaller than the infarction groups. While this disparity reflects contemporary clinical reality in the high-sensitivity troponin era—where strictly adjudicated unstable angina is increasingly scarce—it limits statistical power for certain subgroup analyses. Finally, cause-specific mortality was not adjudicated in our registry database. Over a 10-year observation window, non-cardiovascular causes of death inevitably contribute to cumulative mortality, particularly in an older population with substantial chronic comorbidities. This lack of cause-specific adjudication introduces potential non-cardiac confounders that may obscure the direct longitudinal impact of the index acute coronary event. Given the observational and retrospective nature of our registry, the reported long-term outcome comparisons reflect statistical associations rather than direct cause-and-effect relationships. Accordingly, our findings should be interpreted as highlighting long-term clinical vulnerability associated with baseline comorbidity patterns rather than establishing direct therapeutic or pathophysiological causality.

## 5. Conclusions

In conclusion, while patients with unstable angina experience favorable acute stabilization, their long-term crude mortality approaches that observed in STEMI. However, given substantial baseline cohort imbalances, direct unadjusted comparisons must be interpreted with extreme caution; multivariable analysis indicates that long-term mortality is primarily driven by age, renal function, and comorbidity burden rather than acute disease subtype. These findings highlight that biomarker-negative acute chest pain should not be equated with a benign long-term prognosis, reinforcing the need for comprehensive secondary prevention.

## Figures and Tables

**Figure 1:**
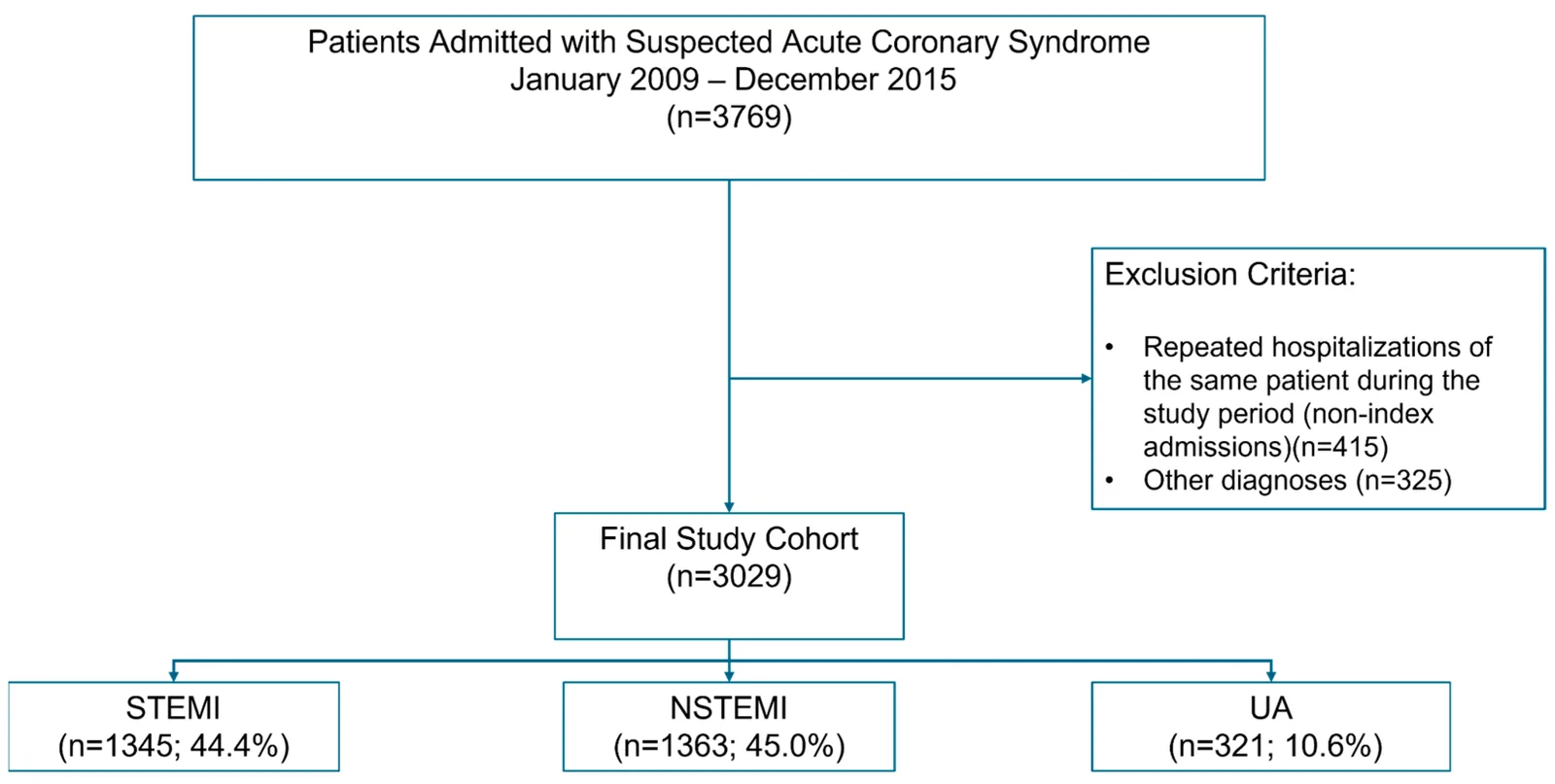
Study flowchart and patient selection. NSTEMI, non-ST-segment elevation myocardial infarction; STEMI, ST-segment elevation myocardial infarction; UA, unstable angina.

**Figure 2:**
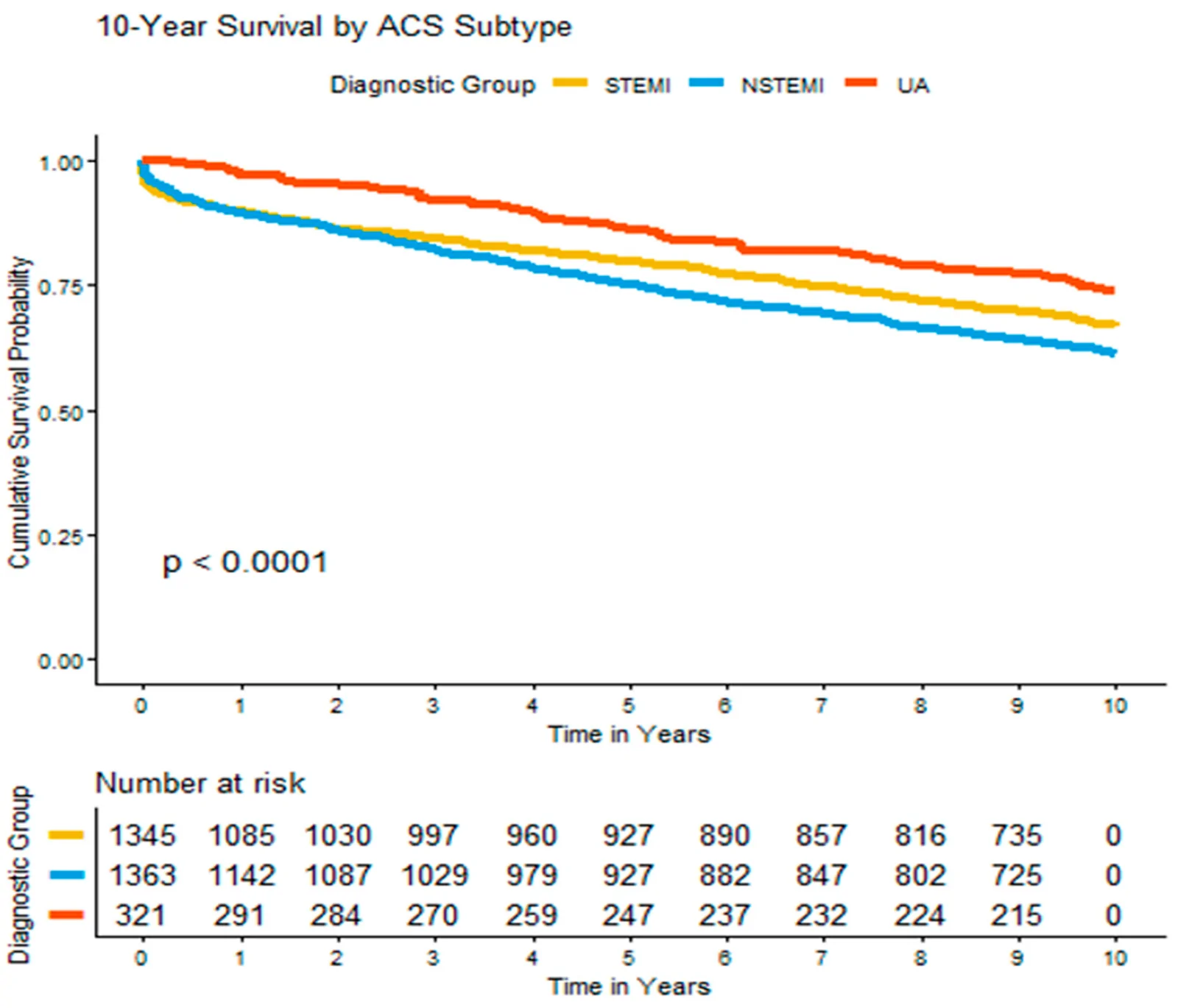
**Kaplan–Meier survival estimates for 10-year all-cause mortality according to acute coronary syndrome subtype**. Cumulative survival probabilities across the three diagnostic groups were compared using the log-rank test. The number at risk table below the graph indicates the cohort size remaining under observation at each yearly interval. Note: The number at risk drops to 0 at the end of Year 10 (day 3652.5) because follow-up was administratively censored at this predefined cutoff; this zero reflects the planned exit of all surviving patients from observation, not mortality events. NSTEMI, non-ST-segment elevation myocardial infarction; STEMI, ST-segment elevation myocardial infarction; UA, unstable angina.

**Figure 3:**
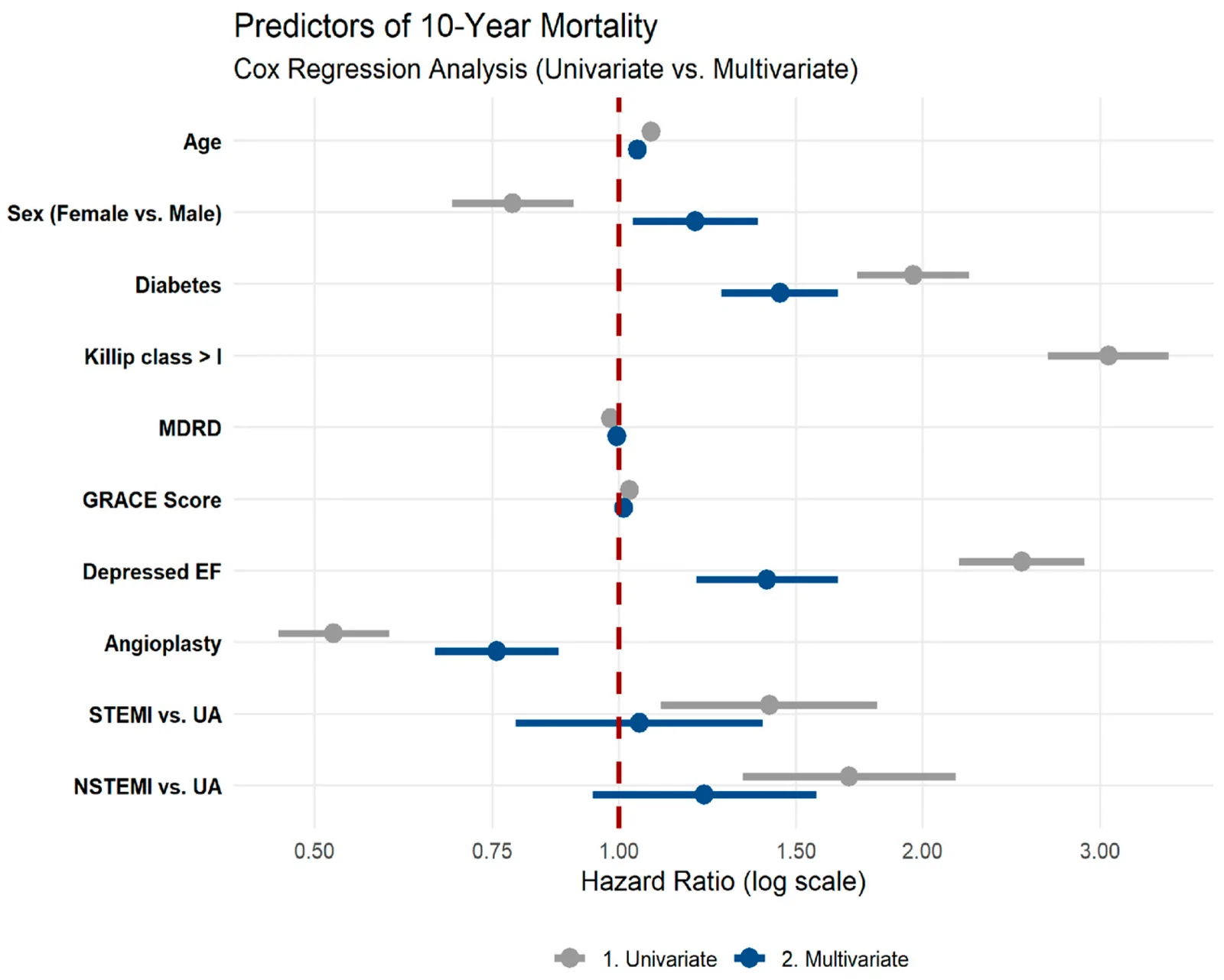
**Forest plot detailing the univariate and multivariate Cox regression analysis for predictors of 10-year mortality**. Point estimates represent Hazard Ratios (HR) and error bars indicate 95% confidence intervals (CI). Gray markers correspond to the unadjusted univariate analysis, while blue markers represent the adjusted multivariate model. The vertical dashed red line at HR = 1.0 indicates the threshold of no effect. Abbreviations: CI, confidence interval; EF, ejection fraction; GRACE, Global Registry of Acute Coronary Events; HR, hazard ratio; MDRD, Modification of Diet in Renal Disease; NSTEMI, non-ST-segment elevation myocardial infarction; STEMI, ST-segment elevation myocardial infarction; UA, unstable angina. Notably, clinical presentation with either STEMI or NSTEMI was associated with a higher crude mortality risk compared to unstable angina. Other factors significantly increasing the unadjusted risk of death included older age, diabetes mellitus, Killip class > I at admission, a higher GRACE score, and a depressed left ventricular ejection fraction. Conversely, female sex, a higher estimated glomerular filtration rate (eGFR), and the performance of percutaneous coronary intervention were associated with a lower crude mortality risk. However, in the multivariate Cox regression analysis, age, female sex, diabetes mellitus, lower eGFR, higher GRACE score, depressed LVEF, and the performance of percutaneous coronary intervention emerged as the independent predictors of 10-year mortality.

**Table 1:** Baseline Clinical and Demographic Characteristics.

	Total Population	STEMI	NSTEMI	UA	*p*-Value
	(N = 3029)	(N = 1345)	(N = 1363)	(N = 321)	
**Demographics**					
Sex male	2224 (73.4%)	1018 (75.7%) ^a^	981 (72.0%) ^a^	225 (70.1%) ^a^	0.033
Age (years)	65.0 [56.0–75.0]	62.0 [52.5–73.0] ^a^	67.0 [57.0–76.0] ^b^	66.0 [59.0–75.0] ^ab^	<0.001
BMI (kg/m^2^)	27.10 [24.82–29.76]	26.93 [24.64–29.66] ^a^	27.16 [24.79–30.04] ^a^	27.31 [25.55–29.74] ^a^	0.775
**History of Cardiovascular Disease**					
Angina > 1 month ago	487 (16.1%)	99 (7.4%) ^a^	265 (19.4%) ^b^	123 (38.3%) ^c^	<0.001
Previous Myocardial Infarction	553 (18.3%)	153 (11.4%) ^a^	299 (21.9%) ^b^	101 (31.5%) ^c^	<0.001
Previous Heart Failure	98 (3.2%)	25 (1.9%) ^a^	63 (4.6%) ^b^	10 (3.1%) ^ab^	<0.001
Previous Stroke	194 (6.4%)	64 (4.8%) ^a^	109 (8.0%) ^b^	21 (6.5%) ^ab^	0.003
Previous Peripheral Artery Disease	252 (8.3%)	72 (5.4%) ^a^	156 (11.4%) ^b^	24 (7.5%) ^ab^	<0.001
Previous Renal Failure	238 (7.9%)	69 (5.1%) ^a^	154 (11.3%) ^b^	15 (4.7%) ^a^	<0.001
Previous Pulmonary Disease	424 (14.0%)	146 (10.9%) ^a^	229 (16.8%) ^b^	49 (15.3%) ^ab^	<0.001
Previous Angioplasty	313 (10.3%)	92 (6.8%) ^a^	145 (10.6%) ^b^	76 (23.7%) ^c^	<0.001
Previous Cardiac Surgery	31 (1.0%)	8 (0.6%) ^a^	16 (1.2%) ^ab^	7 (2.2%) ^b^	0.030
**Cardiovascular Risk Factors**					
Current smoker	998 (32.9%)	543 (40.4%) ^a^	396 (29.1%) ^b^	59 (18.4%) ^c^	<0.001
Diabetes	975 (32.2%)	347 (25.8%) ^a^	512 (37.6%) ^b^	116 (36.1%) ^b^	<0.001
Hypertension	2003 (66.1%)	779 (57.9%) ^a^	986 (72.3%) ^b^	238 (74.1%) ^b^	<0.001
Hypercholesterolemia	1635 (54.0%)	633 (47.1%) ^a^	785 (57.6%) ^b^	217 (67.6%) ^c^	<0.001
**Prior Treatment**					
Prior Aspirin	938 (31.0%)	283 (21.0%) ^a^	477 (35.0%) ^b^	178 (55.5%) ^c^	<0.001
Prior P2Y12 inhibitors	330 (10.9%)	82 (6.1%) ^a^	178 (13.1%) ^b^	70 (21.8%) ^c^	<0.001
Prior Beta-blockers	703 (23.2%)	212 (15.8%) ^a^	357 (26.2%) ^b^	134 (41.7%) ^c^	<0.001
Prior ACE Inhibitors	793 (26.2%)	290 (21.6%) ^a^	397 (29.1%) ^b^	106 (33.0%) ^b^	<0.001
Prior Oral Hypoglycemics	526 (17.4%)	195 (14.5%) ^a^	262 (19.2%) ^b^	69 (21.5%) ^b^	<0.001
Prior Statins	1234 (40.7%)	422 (31.4%) ^a^	600 (44.0%) ^b^	212 (66.0%) ^c^	<0.001

Categorical variables are presented as absolute frequencies and percentages, n (%). Continuous variables are expressed as median [interquartile range, Q1–Q3]. Statistical comparisons across the three acute coronary syndrome groups were performed using the Pearson Chi-square test for categorical variables and the Kruskal–Wallis test for continuous variables. A Bonferroni correction was applied for post hoc multiple pairwise comparisons; different superscript letters (a, b, c) within the same row denote subgroups that differ significantly from each other at the *p* < 0.05 level. Subgroups sharing the same letter do not show a statistically significant difference. A two-sided *p*-value < 0.05 was considered statistically significant. Abbreviations: ACE, angiotensin-converting enzyme; BMI, body mass index; NSTEMI, non-ST-segment elevation myocardial infarction; STEMI, ST-segment elevation myocardial infarction; UA, unstable angina.

**Table 2:** Clinical Presentation, Risk Stratification, and In-Hospital Management.

	Total Population	STEMI	NSTEMI	UA	*p*-Value
	(N = 3029)	(N = 1345)	(N = 1363)	(N = 321)	
**Predominant Symptom**					
Chest pain	2753 (90.9%)	1222 (90.9%) ^a^	1220 (89.5%) ^a^	311 (96.9%) ^b^	<0.001
Dyspnea	125 (4.1%)	41 (3.0%) ^a^	79 (5.8%) ^b^	5 (1.6%) ^a^	<0.001
Recovered cardiac arrest	43 (1.4%)	30 (2.2%) ^a^	13 (1.0%) ^b^	0 (0.0%) ^b^	<0.001
**Admission Clinical Characteristics**					
Heart Rate at admission (bpm)	74.0 [63.0–86.0]	73.0 [61.0–86.0] ^a^	75.0 [65.0–88.0] ^b^	70.0 [61.0–80.0] ^c^	<0.001
Systolic BP at admission (mmHg)	135.0 [118.0–155.0]	129.0 [111.5–145.0] ^a^	140.0 [121.0–160.0] ^b^	138.0 [123.0–158.0] ^b^	<0.001
Admission Killip > I	525 (17.3%)	279 (20.7%) ^a^	238 (17.5%) ^a^	8 (2.5%) ^b^	<0.001
Initial ECG Rhythm					
AF or Flutter	228 (7.5%)	84 (6.2%) ^a^	124 (9.1%) ^b^	20 (6.2%) ^ab^	<0.001
LBBB	127 (4.2%)	115 (8.6%) ^a^	0 (0.0%) ^b^	12 (3.7%) ^c^	<0.001
Pacemaker	41 (1.4%)	0 (0.0%) ^a^	36 (2.6%) ^b^	5 (1.6%) ^b^	<0.001
**Laboratory Findings**					
Glucose at admission (mg/dL)	128.5 [102.0–170.5]	137.0 [108.0–180.0] ^a^	125.5 [101.0–168.5] ^b^	111.0 [93.0–150.0] ^c^	<0.001
eGFR by MDRD (mL/min/1.73 m^2^)	82.46 [61.82–102.79]	85.65 [65.22–107.05] ^a^	79.27 [57.87–100.78] ^b^	84.05 [66.18–102.47] ^a^	<0.001
LDL Cholesterol (mg/dL)	99.30 [79.57–123.00]	101.40 [81.65–126.00] ^a^	99.00 [79.00–123.00] ^a^	94.00 [75.00–114.00] ^a^	0.086
**Risk Stratification**					
GRACE Score	106.80 [86.97–130.07]	113.30 [95.20–134.10] ^a^	107.67 [85.50–131.25] ^b^	89.90 [73.70–106.20] ^c^	<0.001
**In-Hospital Procedures and Interventions**					
Ergometry performed (Yes)	257 (8.5%)	41 (3.0%) ^a^	80 (5.9%) ^b^	136 (42.4%) ^c^	<0.001
Moderately or severely depressed LVEF	502 (16.6%)	297 (22.1%) ^a^	190 (13.9%) ^b^	15 (4.7%) ^c^	<0.001
Admission Coronary Angiography	2534 (83.7%)	1221 (90.8%) ^a^	1090 (80.0%) ^b^	223 (69.5%) ^c^	<0.001
Angioplasty	1928 (63.7%)	1102 (81.9%) ^a^	718 (52.7%) ^b^	108 (33.6%) ^c^	<0.001
Bypass	62 (2.0%)	13 (1.0%) ^a^	40 (2.9%) ^b^	9 (2.8%) ^b^	<0.001

Categorical variables are presented as absolute frequencies and percentages, n (%). Continuous variables are expressed as median [interquartile range, Q1–Q3]. Statistical comparisons across the three acute coronary syndrome groups were performed using the Pearson Chi-square test for categorical variables and the Kruskal–Wallis test for continuous variables. A Bonferroni correction was applied for post hoc multiple pairwise comparisons; different superscript letters (a, b, c) within the same row denote subgroups that differ significantly from each other at the *p* < 0.05 level. Subgroups sharing the same letter do not show a statistically significant difference. A two-sided *p*-value < 0.05 was considered statistically significant. Abbreviations: AF, atrial fibrillation; BP, blood pressure; ECG, electrocardiogram; eGFR, estimated glomerular filtration rate; GRACE, Global Registry of Acute Coronary Events; LBBB, left bundle branch block; LDL, low-density lipoprotein; LVEF, left ventricular ejection fraction; MDRD, Modification of Diet in Renal Disease; NSTEMI, non-ST-segment elevation myocardial infarction; STEMI, ST-segment elevation myocardial infarction; UA, unstable angina.

**Table 3:** In-Hospital Clinical Outcomes and Discharge Management.

	Total Population	STEMI	NSTEMI	UA	*p*-Value
	(N = 3029)	(N = 1345)	(N = 1363)	(N = 321)	
**Complications during admission**					
Recurrent angina	147 (4.9%)	36 (2.7%) ^a^	94 (6.9%) ^b^	17 (5.3%) ^b^	<0.001
Reinfarction	77 (2.5%)	30 (2.2%) ^ab^	46 (3.4%) ^b^	1 (0.3%) ^a^	0.005
Stroke	18 (0.6%)	9 (0.7%) ^a^	9 (0.7%) ^a^	0 (0.0%) ^a^	0.342
Mechanical Complications					
Ventricular Septal Defect	6 (0.2%)	6 (0.4%) ^a^	0 (0.0%) ^b^	0 (0.0%) ^ab^	0.023
Mitral Regurgitation	23 (0.8%)	14 (1.0%) ^a^	9 (0.7%) ^a^	0 (0.0%) ^a^	0.132
Wall Rupture	16 (0.5%)	12 (0.9%) ^a^	4 (0.3%) ^a^	0 (0.0%) ^a^	0.038
Recovered Cardiac Arrest	73 (2.4%)	56 (4.2%) ^a^	17 (1.2%) ^b^	0 (0.0%) ^b^	<0.001
2nd or 3rd Degree AV Block	86 (2.8%)	69 (5.1%) ^a^	16 (1.2%) ^b^	1 (0.3%) ^b^	<0.001
**Mechanical and Electrical Support**					
Mechanical Ventilation					
Invasive	109 (3.6%)	75 (5.6%) ^a^	34 (2.5%) ^b^	0 (0.0%) ^c^	<0.001
Non-Invasive	41 (1.4%)	21 (1.6%) ^a^	20 (1.5%) ^a^	0 (0.0%) ^a^	0.083
Electrical Devices					
Permanent Pacemaker	11 (0.4%)	6 (0.4%) ^a^	5 (0.4%) ^a^	0 (0.0%) ^a^	0.490
Temporary Pacemaker	38 (1.3%)	30 (2.2%) ^a^	8 (0.6%) ^b^	0 (0.0%) ^b^	<0.001
**Discharge Status**					
In hospital mortality	118 (3.9%)	73 (5.4%) ^a^	45 (3.3%) ^b^	0 (0.0%) ^c^	<0.001
**Discharge Treatment**					
Aspirin at discharge	2630 (86.8%)	1150 (85.5%) ^a^	1195 (87.7%) ^a^	285 (88.8%) ^a^	0.341
P2Y12 inhibitors at discharge	2292 (75.7%)	1085 (80.7%) ^a^	1013 (74.3%) ^b^	194 (60.4%) ^c^	<0.001
Beta-blockers at discharge	1979 (65.3%)	825 (61.3%) ^a^	922 (67.6%) ^b^	232 (72.3%) ^b^	<0.001
ACE Inhibitors at discharge	1508 (49.8%)	675 (50.2%) ^a^	687 (50.4%) ^a^	146 (45.5%) ^a^	0.263
Oral Hypoglycemics at discharge	522 (17.2%)	156 (11.6%) ^a^	283 (20.8%) ^b^	83 (25.9%) ^b^	<0.001
Statins at discharge	2560 (84.5%)	1106 (82.2%) ^a^	1159 (85.0%) ^a^	295 (91.9%) ^b^	<0.001

Categorical variables are presented as absolute frequencies and percentages, n (%). Continuous variables are expressed as median [interquartile range, Q1–Q3]. Statistical comparisons across the three acute coronary syndrome groups were performed using the Pearson Chi-square test for categorical variables. A Bonferroni correction was applied for post hoc multiple pairwise comparisons; different superscript letters (a, b, c) within the same row denote subgroups that differ significantly from each other at the *p* < 0.05 level. Subgroups sharing the same letter do not show a statistically significant difference. A two-sided *p*-value < 0.05 was considered statistically significant. Abbreviations: ACE, angiotensin-converting enzyme; AV, atrioventricular; NSTEMI, non-ST-segment elevation myocardial infarction; STEMI, ST-segment elevation myocardial infarction; UA, unstable angina.

**Table 4:** Long-Term Clinical Outcomes at 1, 5, and 10 Years.

	Total Population	STEMI	NSTEMI	UA	*p*-Value
	(N = 3029)	(N = 1345)	(N = 1363)	(N = 321)	
**1-Year Follow-up**					
Death	276 (9.1%)	130 (9.7%) ^a^	137 (10.1%) ^a^	9 (2.8%) ^b^	<0.001
MI	129 (4.3%)	33 (2.5%) ^a^	83 (6.1%) ^b^	13 (4.0%) ^ab^	<0.001
CHF	101 (3.3%)	45 (3.3%) ^a^	45 (3.3%) ^a^	11 (3.4%) ^a^	0.993
MACE	437 (14.4%)	188 (14.0%) ^ab^	220 (16.1%) ^b^	29 (9.0%) ^a^	0.004
**5-Year Follow-up**					
Death	610 (20.1%)	250 (18.6%) ^a^	319 (23.4%) ^b^	41 (12.8%) ^c^	<0.001
MI	303 (10.0%)	104 (7.7%) ^a^	169 (12.4%) ^b^	30 (9.3%) ^ab^	<0.001
CHF	231 (7.6%)	81 (6.0%) ^a^	123 (9.0%) ^b^	27 (8.4%) ^ab^	0.011
MACE	916 (30.2%)	375 (27.9%) ^a^	465 (34.1%) ^b^	76 (23.7%) ^a^	<0.001
**10-Year Follow-up**					
Death	948 (31.3%)	392 (29.1%) ^a^	481 (35.3%) ^b^	75 (23.4%) ^a^	<0.001
MI	401 (13.2%)	143 (10.6%) ^a^	219 (16.1%) ^b^	39 (12.1%) ^ab^	<0.001
CHF	337 (11.1%)	120 (8.9%) ^a^	179 (13.1%) ^b^	38 (11.8%) ^ab^	0.002
MACE	1306 (43.1%)	536 (39.9%) ^a^	654 (48.0%) ^b^	116 (36.1%) ^a^	<0.001

Categorical variables are presented as absolute frequencies and percentages, n (%). Statistical comparisons across the three acute coronary syndrome groups were performed using the Pearson Chi-square test. A Bonferroni correction was applied for post hoc multiple pairwise comparisons; different superscript letters (a, b, c) within the same row denote subgroups that differ significantly from each other at the *p* < 0.05 level. Subgroups sharing the same letter do not show a statistically significant difference. A two-sided *p*-value < 0.05 was considered statistically significant. Abbreviations: CHF, congestive heart failure; MACE, major adverse cardiovascular events; MI, myocardial infarction; NSTEMI, non-ST-segment elevation myocardial infarction; STEMI, ST-segment elevation myocardial infarction; UA, unstable angina.

## Data Availability

The data underlying this article will be shared on reasonable request to the corresponding author.

## Abbreviations

The following abbreviations are used in this manuscript:

ACS	Acute Coronary Syndrome
UA	Unstable Angina
NSTEMI	Non-ST-segment Elevation Myocardial Infarction
STEMI	ST-segment Elevation Myocardial Infarction
CHF	Congestive Heart Failure
ECG	Electrocardiogram
LVEF	Left Ventricular Ejection Fraction
eGFR	Estimated Glomerular Filtration Rate
MDRD	Modification of Diet in Renal Disease
GRACE	Global Registry of Acute Coronary Events
MACE	Major Adverse Cardiovascular Events/Major Adverse Cardiac Events
